# Cholinergic Nerve Differentiation of Mesenchymal Stem Cells Derived from Long-Term Cryopreserved Human Dental Pulp In Vitro and Analysis of Their Motor Nerve Regeneration Potential In Vivo

**DOI:** 10.3390/ijms19082434

**Published:** 2018-08-17

**Authors:** Soomi Jang, Young-Hoon Kang, Imran Ullah, Sharath Belame Shivakumar, Gyu-Jin Rho, Yeong-Cheol Cho, Iel-Yong Sung, Bong-Wook Park

**Affiliations:** 1 Department of Oral and Maxillofacial Surgery, College of Medicine, University of Ulsan, Ulsan 44033, Korea; ssooomi@hanmail.net (S.J.); lovenip@mail.ulsan.ac.kr (Y.-C.C.); 2 Department of Dentistry, Gyeongsang National University School of Medicine and Institute of Health Science, Jinju 52727, Korea; omfs00@naver.com; 3 Department of Oral and Maxillofacial Surgery, Changwon Gyeongsang National University Hospital, Changwon 51472, Korea; 4 Department of Theriogenology and Biotechnology, College of Veterinary Medicine and Research Institute of Life Science, Gyeongsang National University, Jinju 52828, Korea; imran.bch@gmail.com (I.U.); sharath0607@gmail.com (S.B.S.); jinrho@gnu.ac.kr (G.-J.R.)

**Keywords:** dental pulp stem cells, tissue cryopreservation, cholinergic nerve differentiation, cell transplantation

## Abstract

The reduction of choline acetyltransferase, caused by the loss of cholinergic neurons, leads to the absence of acetylcholine (Ach), which is related to motor nerve degeneration. The aims of the present study were to evaluate the in vitro cholinergic nerve differentiation potential of mesenchymal stem cells from cryopreserved human dental pulp (hDPSCs-cryo) and to analyze the scale of in vivo motor nerve regeneration. The hDPSCs-cryo were isolated and cultured from cryopreserved dental pulp tissues, and thereafter differentiated into cholinergic neurons using tricyclodecane-9-yl-xanthogenate (D609). Differentiated cholinergic neurons (DF-chN) were transplanted into rats to address sciatic nerve defects, and the scale of in vivo motor nerve regeneration was analyzed. During in vitro differentiation, the cells showed neuron-like morphological changes including axonal fibers and neuron body development, and revealed high expression of cholinergic neuron-specific markers at both the messenger RNA (mRNA) and protein levels. Importantly, DF-chN showed significant Ach secretion ability. At eight weeks after DF-chN transplantation in rats with sciatic nerve defects, notably increased behavioral activities were detected with an open-field test, with enhanced low-affinity nerve growth factor receptor (p75NGFR) expression detected using immunohistochemistry. These results demonstrate that stem cells from cryopreserved dental pulp can successfully differentiate into cholinergic neurons in vitro and enhance motor nerve regeneration when transplanted in vivo. Additionally, this study suggests that long-term preservation of dental pulp tissue is worthwhile for use as an autologous cell resource in the field of nerve regeneration, including cholinergic nerves.

## 1. Introduction

Cholinergic neurons have pivotal roles in cognition, locomotion, and behavioral response, which predominantly use the neurotransmitter, acetylcholine (Ach), for their message delivery [1,2]. The loss of cholinergic neurons results in the reduction of choline acetyltransferase (ChAT) activity, leading to the absence of Ach, which results in motor nerve degeneration, as well as irreversible cognitive decline, as observed in Alzheimer’s disease [3]. Although various therapeutic applications such as cell-based therapies and nerve grafts are being practiced for the treatment of a wide variety of neurological disorders, a novel treatment method for the loss of cholinergic neurons is yet to be developed [4,5]. Recently, various stem-cell-based therapies were proposed as an alternative treatment modality to relieve the pathophysiology of cholinergic nerve disorders, including Alzheimer’s disease and motor nerve disorders [3,5,6]. One of the most feasible and effective stem cell sources for treating neurological disorders is that of neural stem cells; however, these are difficult to harvest from adults [7]. Embryonic stem cells and induced pluripotent stem cells were also studied for their higher in vitro and in vivo neuronal differentiation potential; however, these have many drawbacks for clinical application in humans, including ethical concerns, difficulty in isolating and cultivating autologous cells, and economical demerits [8,9].

Mesenchymal stem cells (MSCs) from various sources were recently revealed as an alternative therapeutic agent, owing to their high stemness, self-renewal, and multi-lineage differentiation properties [10,11,12,13,14]. MSCs from bone marrow, skin, umbilical cord, muscle, and dental tissues successfully differentiate into neuron- or Schwann cell-like cells [10,11,12,13,14,15,16,17,18,19]. In particular, dental pulp-derived stem cells (DPSCs) displayed MSC characteristics and powerful neurogenic differentiation potential in vitro and in vivo [15,16,17,18,19]. One hypothesis explaining this ability is that the origin of dental pulp from the neural crest allows DPSCs to easily differentiate into neuronal cells compared to other cell lines [20]. Moreover, because of its immune modulatory effects, DPSCs can activate the microglia/astroglia in the host microenvironment, and thereby enhance Wallerian degeneration and macrophage invasion. This, in turn, accelerates peripheral nerve regeneration [15,19]. However, the differentiation and regeneration of cholinergic neurons from stem cells is yet to be broadly studied.

Interestingly, in our previous studies, a long-term tissue cryopreservation method was developed using slow-programmed cryopreservation protocols for human dental tissues and Wharton’s jelly for subsequent use as an autologous stem-cell resource [21,22]. More specifically, dental follicle tissues from extracted wisdom teeth were cryopreserved for more than a year, and stem cells were successfully isolated from the cryopreserved tissues thereafter [21,23]. Stem cells from cryopreserved dental follicles (hDFCs-cryo) displayed the same MSC characteristics as stem cells isolated from fresh dental follicles (hDFCs-fresh) [21]. Moreover, hDFCs-cyro and hDFCs-fresh revealed the same in vivo bone regeneration potential and immunomodulatory effects following in vivo transplantation [23].

In the present study, dental pulp stem cells were similarly isolated from long-term (more than a year) cryopreserved human dental pulp tissues harvested from extracted wisdom teeth (hDPSCs-cryo), and thereafter characterized and compared with hDPSCs from fresh dental pulp (hDPSCs-fresh). In addition, hDPSCs-cryo were differentiated into cholinergic neurons in vitro using the tricyclodecane-9-yl-xanthogenate (D609) induction protocol. Differentiated hDPSC cells were evaluated on the basis of their morphological changes, their expression of cholinergic neuron-specific markers, and their ability to secrete acetylcholine. Finally, the differentiated cholinergic neurons (DF-chN) were transplanted into experimental rats with motor nerve defects, and the functional motor nerve regeneration scale was comparatively analyzed with non-cell-transplanted controls using histological and immunohistochemical analysis of the regenerated nerve fibers.

## 2. Results

### 2.1. Evaluation of Cell Morphology, Survivability, Proliferation Rate and Cell Cycle Analysis

**Figure 1:**
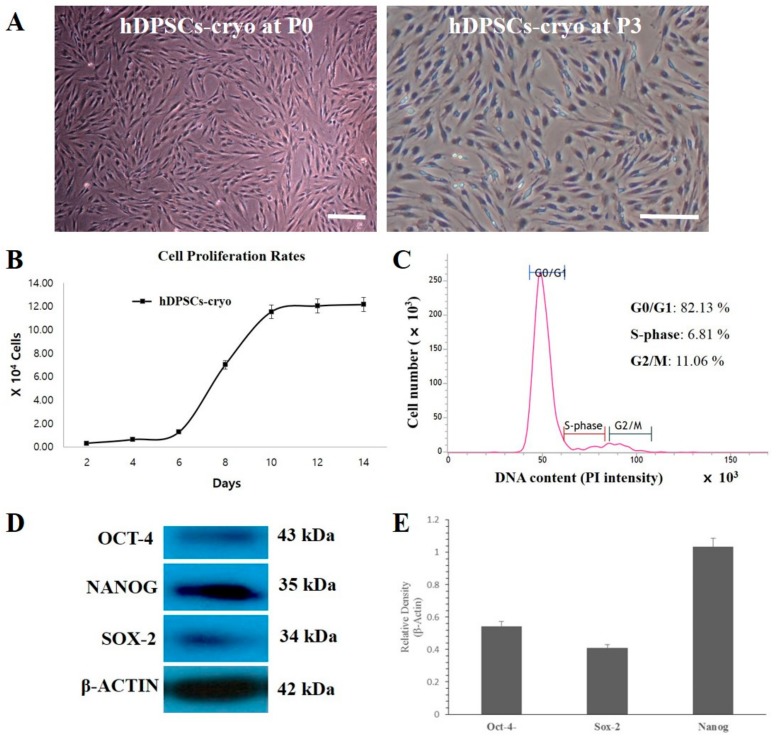
Morphologies, culture characteristics, and cell proliferation rates of cryopreserved human dental pulp tissues harvested from extracted wisdom teeth (hDPSCs-cryo). (**A**) Cell morphologies of hDPSCs-cryo at passage 0 (P0) and passage 3 (P3) showing homogeneous plate adherent fibroblast-like morphology (scale bar = 100 µm); (**B**) Cell proliferation rate by population doubling time (PDT) of hDPSCs-cryo produced normal cell proliferation curvatures. Data represent the mean ± SD of three independent experiments; (**C**) Cell cycle analysis of hDPSCs-cryo showed normal DNA content in the gap 0/1 (G0/G1), synthesis (S), or gap 2/mitotic (G2/M) phases of the cell cycle; (**D**) Western blot analysis revealed positive protein expression of the pluripotent markers, octamer-binding transcription factor 4 (Oct4), Nanog, and sex determining region Y-box 2 (Sox2); and (**E**) The relative quantification of pluripotent marker expressions (**D**) to the control protein (β-actin) revealed relatively higher expression of Nanog over Oct4 or Sox2. Data represent the mean ± SD of three independent experiments.

The cell survival rate of cryopreserved dental pulp tissue-derived MSCs was found to be 79.5 ± 3.0% as evaluated by propidium iodine (PI) and Hoechst 33342 staining, similar to our previous studies [21,23]. Fibroblast-like spindle colonies were observed within the first week of culture, which became homogeneous at passage 3 upon sub-culturing (Figure 1A). The population doubling time (PDT) graph and cell cycle analysis of hDPSCs-cyro showed normal cell proliferation curvatures and normal DNA content in the gap 0/1 (G0/G1), synthesis (S), or gap 2/mitotic (G2/M) phases of the cell cycle (Figure 1B,C). Furthermore, Western blot analysis revealed strong expressions of pluripotency marker proteins such as octamer-binding transcription factor 4 (Oct4), sex determining region Y-box 2 (Sox2), and Nanog in hDPSCs-cryo at passage 3 (Figure 1D,E).

### 2.2. Cell Surface Marker Analysis and Lineage Specific Differentiation of hDPSCs-Cryo

**Figure 2:**
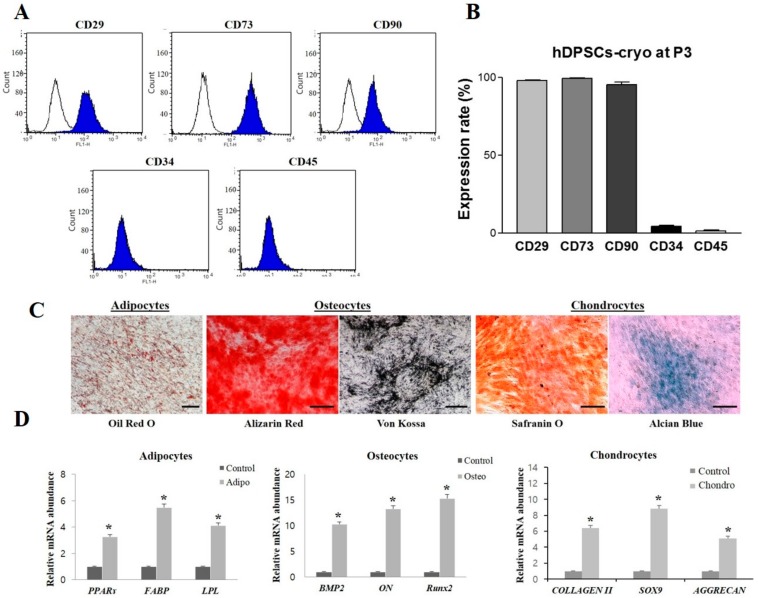
Characterization of hDPSCs-cryo at passage 3. (**A**,**B**) Fluorescence-activated cell sorting (FACS) analysis for hematopoietic and mesenchymal stem cell (MSC) markers revealed high MSC-marker expression (cluster of differentiation (CD)29, CD73, and CD90), whereas hematopoietic markers (CD34 and CD45) were almost negatively expressed; (**C**) hDPSCs-cryo showed successful in vitro differentiation potential to mesenchymal lineage, as confirmed by lineage specific staining (Oil red O for adipocytes, Alizarin red and von Kossa for osteocytes, and Safranin O and Alcian blue for chondrocytes; scale bar = 100 µm); and (**D**) The messenger RNA (mRNA) levels of lineage-specific genes were analyzed using quantitative real-time PCR (RT-qPCR) with the 2^−^^ΔΔ*C*t^ method using tyrosine 3-monooxygenase/tryptophan 5-monooxygenase activation protein, zeta polypeptide (*YWHAZ*) for normalization. Expression levels are expressed as fold change relative to the control (undifferentiated hDPSCs-cryo) [peroxisome proliferator-activated receptor gamma-2 (*PPARγ2*), lipoprotein lipase (*LPL*), and fatty-acid binding protein 4 (*FABP4*), adipocyte-specific; bone morphogenetic protein 2 (*BMP2*), runt-related transcription factor 2 (*Runx2*), and osteonectin (*ON*), osteocyte-specific; and sex determining region Y-box 9 (*SOX9*), cartilage-specific proteoglycan core protein (*AGGRECAN*); and type II collagen (*COLLAGEN II*); chondrocyte-specific]. Data represent the mean ± SD of three independent experiments. * denotes significant differences, *p* < 0.05.

In fluorescence-activated cell sorting (FACS) analysis, hDPSCs-cryo at passage 3 showed increased expression of MSC markers (cluster of differentiation (CD)29, CD73, and CD90), while there was near-negative expression of hematopoietic markers (CD34 and CD45) (Figure 2A,B). In addition, hDPSCs-cryo showed successful in vitro differentiation potential into all the mesenchymal lineages, such as osteocytes, adipocytes, and chondrocytes under specific induction media for 21 days, as confirmed by specific cytochemical staining (Oil red O for adipocytes, Alizarin red and von Kossa for osteocytes, and Safranin O and Alcian blue for chondrocytes) (Figure 2C). In addition, RT-qPCR showed differentiated cells possessed increased messenger RNA (mRNA) levels of lineage-specific genes: fatty-acid binding protein (*FABP*), lipoprotein lipase (*LPL*), and peroxisome proliferator-activated receptor gamma (*PPARγ*) in adipocytes; osteonectin (*ON*), runt-related transcription factor 2 (*RUNX2*), and bone morphogenetic protein 2 *(BMP2*) in osteocytes; and type II collagen (*COLLAGEN II*), sex determining region Y-box 9 (*SOX9*), and cartilage-specific proteoglycan core protein (*AGGRECAN)* in chondrocytes. All differentiated cells had significantly higher mRNA levels of lineage-specific genes, compared to those in undifferentiated hDPSCs-cryo (control; *p* < 0.05) (Figure 2D). These results suggest that stem cells from cryopreserved dental pulps possess MSC characteristics.

### 2.3. Cholinergic Neuronal Differentiation of hDPSCs-Cryo

**Figure 3:**
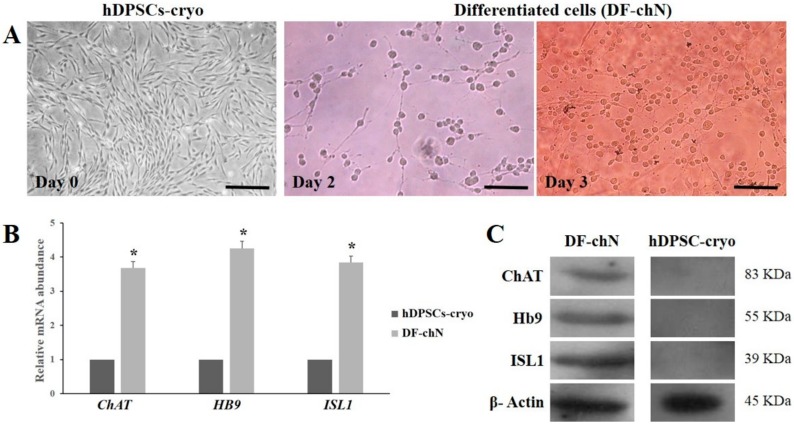
Morphological changes during cholinergic neuronal differentiation of hDPSCs-cryo and expression levels of cholinergic neuron-specific markers. (**A**) Morphology of hDPSCs-cryo (day 0) changed to neuron-like cells, possessing neuronal body and axonal fibers, after the induction time passed (day 2 and day 3) (scale bar = 50 µm); (**B**) Differentiated cholinergic neurons (DF-chN) at day 3 showed increased mRNA levels of cholinergic-specific genes, choline acetyltransferase (*ChAT*), homeobox HB9 (*HB9*), and insulin gene enhancer protein ISL-1 (*ISL1*), using RT-qPCR. Data represent the mean ± SD of three independent experiments (* denotes significant differences, *p* < 0.05); and (**C**) Cholinergic marker protein expression using Western blot analysis in both differentiated neurons (DF-chN) and undifferentiated control (hDPSCs-cryo). DF-chN after tricyclodecane-9-yl-xanthogenate (D609) treatment in hDPSC-cryo showed increased expression levels of cholinergic-specific proteins, ChAT, HB9, and ISL1, whereas the expression of these marker proteins in undifferentiated hDPSCs-cryo was undetectable.

**Figure 4:**
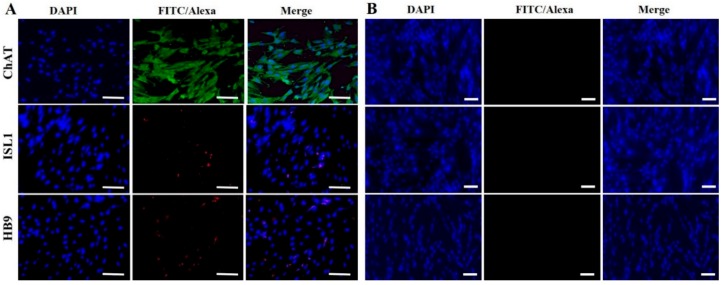
Immunocytochemical analysis of DF-chN (**A**) and undifferentiated hDPSCs-cryo (**B**) for cholinergic-specific proteins. Similar to the Western blot analysis, DF-chN with D609 treatment revealed strong expression of cholinergic-specific proteins, ChAT, HB9, and ISL1, whereas the same proteins were not expressed in undifferentiated hDPSCs-cryo (Scale bar = 50 µm).

To evaluate the cholinergic neuronal differentiation potential, hDPSCs-cryo at the third passage were induced in neurogenic media for three days. After neuronal induction, cells underwent morphological changes with long axonal and branched dendrites as cholinergic neurons (Figure 3A). However, no such alterations were observed in the control group, which were treated in the same culture medium without D609. Successful differentiation was further confirmed by the ability of differentiated cells to transcribe cholinergic neuron-specific markers, such as choline acetyltransferase (*ChAT*), homeobox HB9 (*HB9*), and insulin gene enhancer protein ISL-1 (*ISL1*), as shown by RT-qPCR. DF-chN showed significantly (*p* < 0.05) higher gene expression in comparison to the untreated control (Figure 3B). Western blot and immunocytochemical analysis substantiated these results, revealing strong positive expression of the cholinergic neuron marker proteins, ChAT, HB9, and ISL1, in DF-chN, whereas absolute negative expression of these proteins was detected in undifferentiated hDPSCs-cryo (Figure 3C and Figure 4).

### 2.4. Quantification of Ach

**Figure 5:**
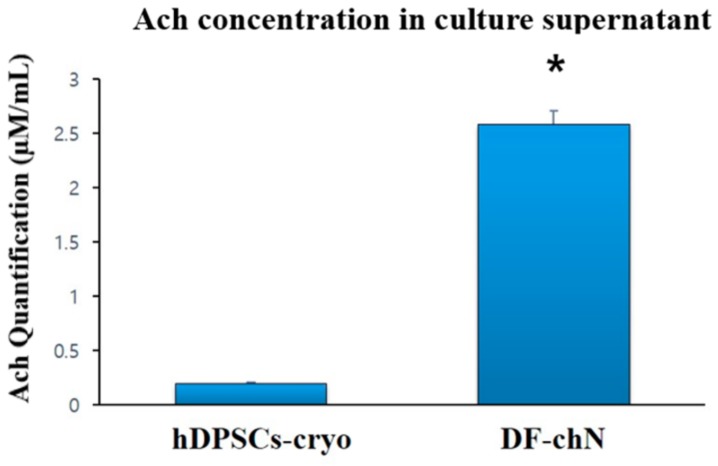
Analysis of acetylcholine (Ach) levels in spent media using a biochemical fluorescent assay. The culture media of DF-chN showed increased Ach levels compared to undifferentiated hDPSCs-cryo, indicating DF-chN could synthesize Ach (mean ± SD of three different experiments; * denotes significant differences, *p* < 0.05).

Ach secretion was measured in culture supernatants of DF-chN and non-differentiated hDPSCs-cryo cells after three days of cholinergic induction. An average of 2.583 µM/mL of Ach secretion was found in DF-chN cells, which was significantly higher than in the non-differentiated hDPSCs-cryo cell group (average 0.198 µM/mL) (Figure 5).

### 2.5. Analysis of In Vivo Regenerated Nerve Fibers

**Figure 6:**
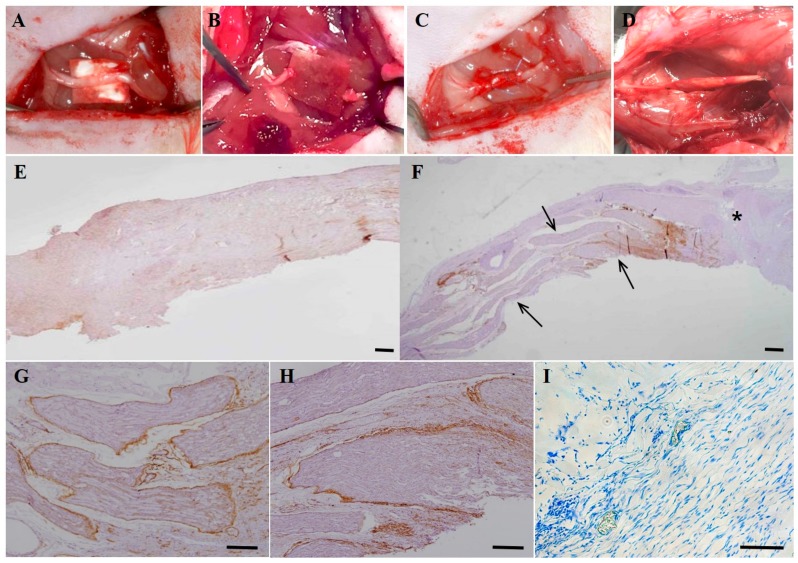
Schematic images and immunohistochemical analysis of in vivo cell transplantation in experimental rats with nerve defects. (**A**) The sciatic nerve was exposed on the thigh of the experimental animal; (**B**) The sciatic nerve was resected and a 5-mm nerve gap developed; (**C**) After tubulization with biodegradable collagen bovine dura mater (Lyoplant^®^) in the nerve gap, a total of 1 × 10^6^ cells (DF-chN) were transplanted into the collagen tube with a 0.1-mL fibrin glue scaffold (Greenplast^®^). The tube was then sealed with sutures; (**D**) Photograph of the experimental nerve at eight weeks after DF-chN transplantation. In the cell transplantation site, the regeneration of nerve defect was detected with continuity of the nerve gap; (**E**–**I**) Histological and immunohistochemical analysis of low-affinity nerve growth factor receptor (p75NGFR) in regenerated motor nerve fibers at eight weeks post cell transplantation. (**E**) In non-cell-transplanted control specimens, regenerated nerve fibers were not detected in abundance and p75NGFR expression was weak. (**F**) In the DF-chN transplanted group, significantly increased regenerating nerve fibers were detected in the nerve gap (arrows). The asterisk (*) indicates the proximal end of the nerve gap. (**G**,**H**) High magnification of generated nerve fibers at the DF-chN graft site. High expression of p75NGFR was detected in newly generated nerve fibers, especially in the perineurium and endoneurium of regenerating nerve fibers. (**I**) Toluidine blue staining of regenerating nerve fibers showed blue-colored positive staining of the myelin sheath, which was detected in abundance in the outer layer of newly generated axonal fibers (Scale bar = 100 µm).

Upon gross inspection at eight weeks after the experiments, the continuity of the resected nerve fiber was obviously identified in the DF-chN transplanted groups (Figure 6D). In non-cell-transplanted control specimen, regenerated nerve fibers were not detected in abundance, and the immunohistochemical (IHC) expression level of low-affinity nerve growth factor receptor (p75NGFR) was weak (Figure 6E). In the DF-chN transplanted group, a remarkable increase in regenerating nerve fibers was detected in the nerve gap (arrows in Figure 6F). In newly generated nerve fibers of the DF-chN grafted site, strong IHC expression of p75NGFR was detected under high magnification, especially in the perineurium and endoneurium (Figure 6G,H). In addition, as shown by Toluidine blue staining, a high number of axonal fibers in the myelin sheath were detected at the DF-chN transplant site (Figure 6I).

**Figure 7:**
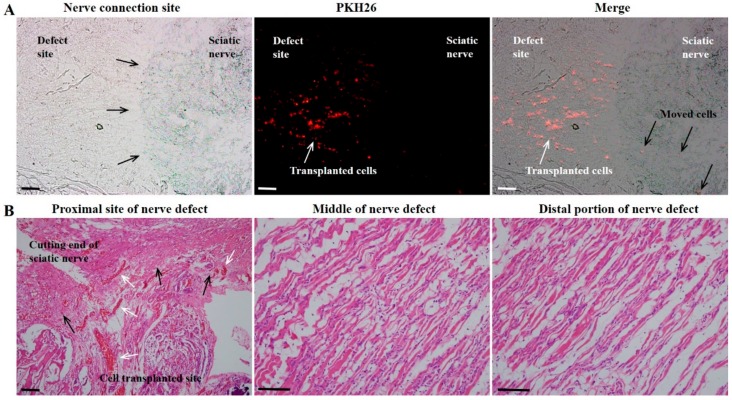
PKH26 expression and histological analysis of the regenerated nerve fibers at eight weeks after cell transplantation. (**A**) In the nerve connection site, the regenerated nerve (defect site) was connected to the cutting end of proximal portion of sciatic nerve (sciatic nerve), black arrows indicating the connection portion. In the same specimen, the fluorescence intensity of pre-transplanted cell-labeling dye PKH26 was remarkably detected in the DF-chN transplanted site (white arrows), demonstrating that the transplanted DF-chN have excellent in vivo adaptability and proliferation abilities. Interestingly, some transplanted cells were also detected in the original sciatic nerve portion (black arrows in Merge), indicating the transplanted DF-chN could move to the original sciatic nerve portion and communicate with the host cells; and (**B**) Histological features of proximal, middle, and distal portions of the nerve defect site. In the proximal portion, black arrows indicate the connection site between the cell-transplanted site and the nerve cutting end. Abundant newly generated blood vessels were detected in this portion (white arrows). In the middle and distal portion of the nerve defect, homogeneous newly generated nerve fibers were evenly detected (Scale bar = 100 µm).

We observed a remarkable fluorescence intensity of PKH26 in the regenerated nerve fibers at the cell-transplanted site, demonstrating that the transplanted DF-chN have excellent in vivo adaptability and proliferation abilities. Moreover, some transplanted cells were also detected in the non-transplanted original sciatic-nerve portion, indicating the transplanted DF-chN could move to the original sciatic nerve portion and communicate with the host cells (Figure 7A). In addition, the histological features of the proximal, middle, and distal portions of regenerated nerves revealed an abundant generation of new blood vessels and homogenous nerve fibers (Figure 7B).

### 2.6. Behavioral Analysis of Animals with In Vivo Cell Transplants

**Figure 8:**
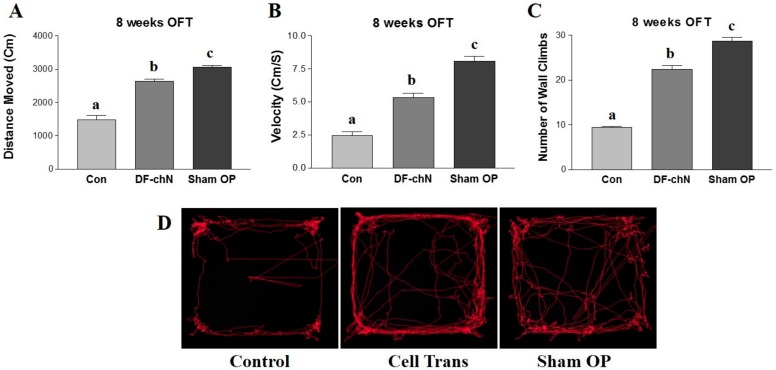
Behavioral analysis of the experimental rats at eight weeks post cell transplantation. The walking ability of experimental rats was evaluated using the open-field test. Over a 10-min period, the velocity (**A**), distance moved (**B**), and number of wall climbs (**C**) were investigated; and (**D**) Representative walking trace of rats at eight weeks post experimental operation. Control, non-cell transplanted negative control; Cell Trans, DF-chN-transplanted group; Sham OP, Sham operation (OP) group. Data represent the mean ± SD of three independent experiments (different letters denote statistically significant differences between groups, *p* < 0.05).

Eight weeks after surgery on the sciatic nerves of experimental rats, their behavioral abilities were evaluated using an open-field test (OFT). In a 10-min period, features such as velocity (cm/s), distance moved (cm), and number of wall-climbing events were investigated. Significant differences (*p* < 0.05) were observed between the cell transplant group (DF-chN) and the negative control group (Con). The distances moved were measured as 1477 ± 211 cm, 2635 ± 124 cm, and 3052 ± 103 cm in the control, DF-chN, and Sham operation (OP) groups, respectively (Figure 8A). The velocities of DF-chN (5.33 ± 0.57 cm/s) and Sham OP (8.09 ± 0.62 cm/s) were also significantly higher than those in the control group (2.48 ± 0.45 cm/s) (Figure 8B). Similarly, the number of wall climbs were recorded as 9, 22, and 29 in the control, DF-chN, and Sham OP groups, respectively (Figure 8C). The representative walking plans of rats after eight weeks of cell transplantation are shown in Figure 8D.

## 3. Discussion

Dental follicles from extracted wisdom teeth were previously cryopreserved using a newly developed tissue cryopreservation protocol [21]. MSCs from these cryopreserved dental follicles were subsequently shown to have immunomodulatory properties that further enhanced osteogenesis under in vivo conditions [23]. In light of these observations, the present study demonstrated that long-term preserved dental pulp tissues stored under the newly developed tissue cryopreservation protocol could safely conserve multipotent stem cells, as shown by the same MSC characteristics as those derived from fresh dental pulp tissues. Both hDPSCs-fresh and hDPSCs-cryo showed the same cell morphology, proliferation rates, cell-surface markers expression, and in vitro differentiation potential as the mesenchymal lineage [15,16,24]. In accordance with previous reports, hDPSCs-cryo used in this study retained their MSC characteristics and were shown to positively express early transcription factors (Nanog, Oct4, Sox2) and MSC markers (CD29, CD73, CD90), and successfully differentiate into the mesenchymal lineage (osteocytes, adipocytes, and chondrocytes). Earlier reports demonstrated that hDPSCs have higher in vitro neurogenic differentiation potential, which not only express neuronal specific markers at high levels, but also possess higher Na^+^ and K^+^ currents along with the expression of synaptic markers [15]. Furthermore, in the present study, hDPSCs-cryo were successfully differentiated into cholinergic neurons (DF-chN) under the tricyclodecane-9-yl-xanthogenate (D609) neuronal induction protocol. When DF-chN was transplanted into the sciatic nerve defects of experimental rats in vivo, a remarkable scale for motor nerve regeneration was detected. These results indicate that long-term cryopreserved dental pulp tissues, using newly developed tissue cryopreservation protocols, could safely preserve their stemness and MSC characteristics without any property change compared to those of fresh dental pulp tissues.

Multiple neuron variants, along with their nature, location, and function, were previously reported: (1) cholinergic neurons play important roles in the physiology of locomotion, cognition, and behavioral response; (2) amino acidergic neurons (r-aminobutyrinergic neurons) are located in Purkinje cells and are involved in neurological disorders, such as Huntington’s disease; and (3) aminergic neurons (dopaminergic, 5-hydroxytryptaminergic, and norepinephrinergic neurons) are located in the basal ganglia and lower brain stem, and show a relationship with Parkinson’s disease, sleep disorders, and sleep/wakefulness cycles [2]. Some methods for neuronal cell differentiation from MSCs were introduced via transgenic technology or using a cocktail induction medium, such as retinoic acid and growth factors, β-mercaptoethanol and butylated hydroxyanisole, or 5-azacytidine and growth factors [25,26,27]. However, the cholinergic nerve differentiation potential of MSCs is yet to be broadly studied, despite the loss of cholinergic neurons resulting in Alzheimer’s disease and motor neuron degeneration [28].

Tricyclodecane-9-yl-xanthogenate (D609), a specific inhibitor of phosphatidylcholine-specific phospholipase C (PC-PLC), was previously observed to differentiate bone marrow MSCs (BMSCs) into neuron-like cells [29,30]. In addition, D609 was shown to induce neuron-like cells possessing cholinergic neuronal characteristics [2,31]; however, the exact mechanism for D609 neurogenic induction in BMSCs was not clear. One suggestion is that D609 treatment in BMSCs inhibits PC-PLC activity, while increasing the levels of heat shock protein 70 (*HSP70*), which activates the transcription regulator B-cell translocation gene 2 (*BTG2*), thereby increasing the levels of functional neuronal specific genes and inducing cholinergic neuronal differentiation [31]. It was also observed that D609 treatment reduced the expression levels of genes involved in mesodermal and endodermal differentiation, whilst increasing the expression levels of genes involved in neuronal differentiation, neuroprotection, and cholesterol synthesis [31]. Cholesterol, in particular, is essential for the synthesis and maintenance of the myelin sheath [32]. Furthermore, in vivo transplantation of differentiated cholinergic neurons in spinal-cord-injured animals promoted neuron functional recovery and protection [2]. It was, therefore, concluded that D609 treatment on BMSCs is a simple and rapid method for cholinergic neuron induction.

The present study similarly demonstrated that stem cells from cryopreserved dental pulp tissues could successfully differentiate into cholinergic neurons using D609 treatment. These differentiated cells showed morphological neuron-like cell characteristics, including neuronal body and axonal fibers, and the positive expression of cholinergic neuronal markers at the mRNA and protein levels. In order to investigate the scale of motor nerve regeneration in vivo, DF-chN were transplanted into experimental rats with sciatic nerve defects using collagen tubulization and a fibrin glue scaffold. In this study, biodegradable bovine collagen dura mater (Lyoplant^®^, Aesculap, Melsungen, Germany) was contoured and sutured to create the tube and bridging for the resected nerve ends, as per previous studies [11,16]. This tubulization provided a mechanical support system for regenerating and remodeling nerve fibers. The resected nerve gap was filled with cells and a fibrin glue scaffold (Greenplast^®^, Green Cross, Yongin, Korea), which was frequently used as a cell delivery system since it rapidly increases the initial stability of grafted cells and permits a stable three-dimensional structure for transplanted cells [11,16].

Eight weeks after cell transplantation, the DF-chN transplanted group showed markedly increased scales of movement, by behavioral analysis, and enhanced nerve fiber regeneration, by histological analysis, when compared to non-cell-transplanted control nerve fibers. In addition, the DF-chN transplanted site showed an abundant regeneration of axonal fibers that were positively stained with Toluidine blue, as well as positive IHC staining for low-affinity nerve growth factor receptor (p75NGFR). The fluorescence intensity of cell-labeling dye PKH26 was abundantly observed at the cell transplanted site, demonstrating that the transplanted DF-chN have excellent in vivo adaptability and proliferation abilities. Moreover, fluorescence intensity of PKH26 dye was also detected in the non-cell-transplanted original sciatic-nerve portion, suggesting that the transplanted DF-chN could move to the non-injured original nerve and communicate with the host cells (Figure 7A). The communication between transplanted cells and host cells might have contributed to the increased peripheral nerve regeneration. Interestingly, histological sections of regenerated nerve fibers of rats revealed no specific increase in the inflammatory response at the cell-transplanted site albeit transplantation being done with a xenogenic cell source. That might be related to the immunomodulatory activity of human dental tissue-derived stem cells [23,33]. Human dental stem cells were reported to show direct suppression of T-cell-mediated immunity and secretion of various soluble factors such as prostaglandin E2, indoleamine 2,3-dioxygenase, transforming growth factor-B, and human leukocyte antigen G5 [33,34]. These immunoregulatory properties of dental stem cells make them a more suitable cell source for immune- and inflammatory-related diseases [23,33,34]. In the present study, hDPSCs-cryo were differentiated into DF-chN, where these differentiated cells might have preserved their immunomodulatory properties, which could have resulted in minimal immune and inflammatory responses upon in vivo transplantation.

In the present study, an OFT was used to analyze functional recovery of the regenerative nerve fibers in experimental animals, whereby walking ability was examined in terms of velocity (cm/s), distance moved (cm), and number of wall climbs completed over a specific time interval. The OFT is an approved locomotor and behavioral assessment method for small animals, including mice and rats, and is widely used to for the behavioral analysis of experimental animals [16,35,36]. The result of the OFT demonstrated that the DF-chN transplanted group showed a significantly improved walking ability (velocity), distance moved, and number of wall climbs completed, as compared to the control group. These results indicated that the in vivo transplantation of DF-chN from hDPSCs-cryo has remarkable therapeutic effects on injured motor nerve fibers.

## 4. Materials and Methods

### 4.1. Chemicals and Media

All chemicals were purchased from Sigma-Aldrich (St. Louis, MO, USA) and all media from Gibco (Invitrogen, Grand Island, NY, USA), unless otherwise specified. All media were adjusted to a pH of 7.4 and to an osmolality of 280 ± 5% mOsm/kg, and filtered using a 0.22-μm filter.

### 4.2. Cryopreservation of Human Dental Pulp Tissues and hDPSCs Isolation

Human dental pulp tissues were collected and stored following approval by the Institutional Review Board of the University Hospital, and with the informed consent of enrolled patients for their tissue donation (GNUH IRB-2012-09-004, 01, 10, 2012). Dental pulp tissues harvested from wisdom teeth extracted (*n* = 8, four for tissue cryopreservation, and four for fresh dental pulp harvesting; aged 14–19 years, average: 18.5 years old) at the Department of Oral and Maxillofacial Surgery at Changwon Gyeongsang National University Hospital were cryopreserved as previously described [21,23]. Briefly, after being collected from the extracted wisdom tooth using a sterile scalpel (Figure 1A), the dental pulp tissue from a single donor was minced into 1–3-mm^2^ explants and tissue segments, and was placed into a 1.8 mL cryovial (Thermo scientific, Roskilde, Denmark) containing 1 mL of cryoprotectant, consisting of 0.05 M glucose, 0.05 M sucrose, and 1.5 M ethylene glycol in phosphate-buffered saline (PBS) (osmolality: 1900 mOsm/kg). A programmed slow-freezing protocol was then applied for cryopreservation. Briefly, cryovials were equilibrated for 30 min at 1 °C, then cooled at −2 °C/min to −9.0 °C, then from −9.0 °C to −9.1 °C, and held for 5 min. Cryovials were then further cooled at −0.3 °C/min to −40 °C, then at −10 °C/min to −140 °C (Figure 1B), and stored in liquid nitrogen for more than a year. Before use, cryopreserved dental pulp tissues were thawed in a circulating water bath at 37 °C for 1 min.

Fresh dental pulp tissues from the other four donors were rinsed with Dulbecco’s phosphate-buffered saline (DPBS) containing 1% penicillin-streptomycin (Pen-Strep), and minced into 1–3-mm^2^ explants for immediate hDPSC isolation. Tissue explants from fresh and cryopreserved dental pulp were treated for hDPSC isolation and cultivation, as previously described [15,16,21,23]. Briefly, samples were rinsed with DPBS containing 1% Pen-Strep; the tissues were chopped into pieces, and then digested with 1 mg/mL collagenase type I for 40 min at 37 °C with gentle agitation. Following digestion, cell suspensions were filtered through a 100- and 40-µm nylon cell strainer (BD Falcon, Franklin Lake, NJ, USA) in order to harvest single-cell suspensions. Further digestion was prevented by adding advanced Dulbecco’s modified Eagle’s medium (ADMEM) supplemented with 10% fetal bovine serum (FBS). A total of 1 × 10^5^ cells were initially seeded into a 25 T-flask (Nunc^TM^, Roskilde, Denmark) containing ADMEM supplemented with 10% FBS and 1% Pen-Strep. Culture dishes were kept at 37 °C in a humidified incubator containing 5% CO_2_ in air. At 80–90% confluence, cells were dissociated with 0.25% (*w*/*v*) trypsin EDTA solution and sub-cultured until passage 3. MSCs from hDPSCs at the third passage were used for the remaining experiments.

### 4.3. Analysis of Pluripotent Markers Using Western Blot

For the evaluation of pluripotent markers, Western blotting was performed. Briefly, protease inhibitor-containing RIPA buffer (PIERCE, Rockford, IL, USA) was used to prepare protein lysate from all experimental groups. Total protein was quantified using the Microplate BCA protein assay kit (PIERCE). A 20 µg protein sample from each experimental group was separated using 12% sodium dodecyl sulfate polyacrylamide gel electrophoresis (SDS-PAGE; Mini Protein, BioRad^TM^, Hercules, CA, USA), and thereafter transferred onto a polyvinylidene difluoride membrane (PVDF, BioRad^TM^). PVDF membranes were blocked with 5% bovine serum albumin (BSA) in Tris-buffered saline (1× TBS) for 1 h at 20 °C, followed by washing with 0.1% TBS-Tween (TBST). The membranes were then incubated with primary antibodies, including goat anti-Oct4 (1:200, 43–50 kDa, Santa Cruz Biotechnology, Inc., Dallas, TX, USA), mouse anti-Nanog (1:200, 35 kDa, Santa Cruz), rabbit anti-Sox2 (1:200, 34 kDa, Santa Cruz), and rabbit anti-β-actin (1:1000, Cell signaling, Danvers, MA, USA) overnight at 4 °C. After washing with 0.1% TBST, membranes were incubated with horseradish peroxidase (HRP)-conjugated goat anti-mouse (1:1000, Santa Cruz), rabbit anti-goat (1:1000, Santa Cruz), and goat anti-rabbit (1:1000, Santa Cruz) secondary antibodies for 1 h at 20 °C. Immunoreactivity was detected by enhanced chemiluminescence (ECL; Pierce^TM^ ECL Plus, thermo Scientific^TM^, Waltham, MA, USA). The membranes were then exposed to X-ray film in the dark, and were developed, with the resulting bands subsequently analyzed.

### 4.4. Analysis of Cell Survival and Proliferation Rates

Immediately following cell isolation from fresh and cryopreserved dental pulp tissue, cells were stained with propidium iodine (PI; Sigma-Aldrich, St. Louis, MI, USA) and Hoechst 33342 (Sigma-Aldrich, St. Louis, MI, USA), as previously described [21]. To calculate the cell survival rate in each group (fresh pulp vs. cryopreserved pulp), the proportion of PI positive cells (dead cells) to Hoechst 33342 stained cells (all of the live and dead cells) were calculated under a fluorescent microscope (Nikon Eclipse Ti-U, Nikon Instrument, Tokyo, Japan) (Figure 1C,D).

The morphologies of hDPSCs-fresh and hDPSCs-cryo cells were observed and analyzed during cell culture under a light microscope. Images were captured using a Nikon DIAPHOT 300 (Nikon Instrument) (Figure 2A). The proliferation rates of hDPSCs-fresh and hDPSCs-cryo were analyzed by population doubling time (PDT), as previously described [22]. Briefly, MSCs were seeded in triplicate at a density of 2 × 10^3^ cells per well using a 24-well culture plate. Cell numbers were determined at two-day intervals up to a total of 14 days. The PDT of MSCs was calculated using the formula PDT = *t* (log2)/(log*N_t_ –* log*N*_0_), where *t* represents culture time, and *N*_0_ and *N_t_* are the initial and final hDPSC numbers before and after seeding, respectively (Figure 2B).

### 4.5. Flow Cytometry and DNA Content

Dental pulp stem cells were analyzed for the expression of cell-surface markers and DNA content by fluorescence-activated cell sorting (FACS; BD FACSCalibur, Becton Dickson, Franklin Lakes, NJ, USA) in three independent experiments, as previously described [21,23]. Briefly, third-passage hDPSCs-cryo at 80% confluence were fixed with 3.7% formaldehyde for 30 min. The cells were then washed twice with DPBS and labelled (1 × 10^5^ cells per marker) by incubating with fluorescein isothiocyanate-conjugated CD34 (BD Pharmingen, San Jose, CA, USA, FITC Mouse Anti-Human CD34), CD45 (Santa Cruz Biotechnology, FITC Mouse Anti-Human CD45), CD90 (BD Pharmingen, FITC Mouse Anti-Human CD90) and unconjugated CD73 (Santa Cruz Biotechnology, Mouse monoclonal), and unconjugated CD105 (Santa Cruz Biotechnology, Mouse monoclonal immunoglobulin G (IgG_2a_)) for 30 min. Unconjugated primary antibodies were treated with secondary FITC-conjugated goat anti-mouse IgG (BD Pharmingen) for 30 min in the dark. Mouse IgG_1_ (BD Pharmingen) was used for an isotype-matched negative control. A total of 1 × 10^4^ FITC-labeled cells per sample were acquired, and results were analyzed using the cell Quest Pro software (Becton Dickinson, Franklin Lakes, NJ, USA).

To evaluate DNA content, third-passage hDPSCs-cryo (1 × 10^4^ cells/mL) were fixed in 70% ethanol at 4 °C for 4 h. After washing cells twice with DPBS, cells were stained with 10 mg/mL PI solution for 15 min. The DNA content of each cell was measured and categorized as being either in the G0/G1, S, or G2/M phase of the cell cycle. All experiments were performed in triplicate.

### 4.6. In Vitro Mesenchymal-Lineage Differentiation

In vitro differentiation into mesenchymal lineages (adipocytes, osteocytes, and chondrocytes) was performed using previously described protocols [21,24]. Briefly, third-passage hDPSCs-cryo at 70% confluence were induced to lineage specific growth conditions for 21 days. Adipogenic medium consisted of 1 µM dexamethasone, 10 µM insulin, 100 µM indomethacin, and 500 µM isobutyl methyl xanthine (IBMX). For the confirmation of adipogenesis, differentiated cells were analyzed for the accumulation of lipid droplets by staining with Oil red O solution for 30 min. Osteogenic medium consisted of 50 µM ascorbate-2-phosphate, 10 mM glycerol-2-phosphate, and 0.1 µM dexamethasone. Osteogenesis was confirmed by staining differentiated cells with Alizarin red and von Kossa for the detection of mineralization and calcium deposition, respectively. Chondrogenesis was induced by using commercial chondrogenic medium (StemPro^®^ Osteocyte/Chondrocyte Differentiation Basal Medium; StemPro^®^ Chondrogenesis supplement, Gibco^®^ by life technologies, Grand Island, NY, USA) and differentiation was analyzed by Alcian blue and Safranin O staining. RT-qPCR was used to evaluate the expression level of lineage-specific marker genes.

### 4.7. Cholinergic Neuronal Differentiation of hDPSCs-Cryo

Initially, cell culture plates were coated with Geltrex LDEV (Gibco) for 2 h at 37 °C followed by washing with DPBS prior to the addition of cells. Cholinergic neuronal differentiation of hDPSCs-cryo was conducted as previously described [2] with minor modifications. Briefly, a total of 5 × 10^4^ cells (hDPSCs-cryo) at the third passage were grown on each well of pre-coated six-well plates (Thermo Scientific) with 10% ADMEM. After reaching 70–80% confluence, cells were cultured with induction medium containing 4 µg/mL tricyclodecane-9-yl-xanthogenate (D609) in 10% ADMEM. The control group was maintained in parallel with the same culture medium without the addition of D609. After three days of induction (no medium change), the supernatant was collected for the analysis of Ach concentration by ELISA, and differentiated cells were harvested. The differentiated cells were fixed with 4% paraformaldehyde (PFA) for immunocytochemistry, or harvested for RNA isolation for use in RT-qPCR and in vivo transplantation experiments.

### 4.8. Real Time Quantitative PCR (RT-qPCR) Analysis

Total RNA was isolated from undifferentiated hDPSCs-cyro (control), mesenchymal lineage (adipogenic, osteogenic, and cholinergic) differentiated cells, and DF-chN using an RNeasy Mini Kit (Qiagen, Valencia, CA, USA) and quantified with a spectrophotometer (NanoDrop 1000, Thermo Scientific). A total of 2 µg of RNA was used to synthesize complementary DNA (cDNA) using an Omniscript Reverse Transcription Kit (Qiagen), with 10 µM OligodT primer. The reaction was carried out at 37 °C for 1 h. The cDNA samples were diluted to a uniform concentration of 50 ng/µL and used as a template for PCR amplification. The RT-qPCR reaction was performed using a Rotor Gene Q (Qiagen) with 50 ng of cDNA quantified with Rotor-GeneTM 2X SYBR^®^ Green mix (Qiagen) supplemented with 10 µM specific primer sets (Table 1). The melting curves, amplification curves, and cycle threshold values (*C*_t_) were determined using the Rotor-Gene Q series software (Qiagen). For the normalization of data, tyrosine 3-monooxygenase/tryptophan 5-monooxygenase activation protein, zeta polypeptide (YWHAZ) was used as an internal control. The relative level of target gene expression was calculated according to the 2^−∆∆*C*t^ method.

### 4.9. Immunocytochemistry of DF-chN as Cholinergic Neuron Markers

After cholinergic neuronal induction of hDPSCs-cryo, immunostaining for cholinergic neuron-specific marker proteins was performed using previously described protocols [37]. Briefly, DF-chN were fixed with 3.7% formaldehyde for 40 min, permeabilized with 0.2% Triton X-100 supplemented with 1% BSA, and thereafter blocked with 1% BSA (in DPBS) at 20 °C for 1 h. Cells were then incubated with primary antibody, diluted 1:100, choline acetyltransferase (ChAT; ab68779, Abcam, Cambridge, UK), homeobox HB9 (motor neuron and pancreas homeobox 1, MNX1) (HB9; sc-22542, Santa Cruz), and insulin gene enhancer protein ISL-1 (ISL1; sc101072, Santa Cruz) at 20 °C for 1 h. Following incubation with primary antibodies, cells were washed with DPBS and incubated with 1:200 CruzFluor^TM^ 594 or CruzFluor^TM^ 488 conjugated donkey anti-rabbit or donkey anti-goat or donkey anti-mouse IgG secondary antibodies (Santa Cruz) for 1 h. For nuclear staining, cells were treated with 1 µg/mL 4′,6-diamidino-2-phenylindole (DAPI) for 5 min at 20 °C. Finally, cells were observed under a fluorescence microscope (Nikon Eclipse Ti-U, Nikon Instrument).

### 4.10. Quantification of Ach from the Culture Supernatant of Induced DF-chN

The acetylcholine concentration was measured in the supernatant of induction medium using a previously described protocol [1]. Briefly, an aliquot of culture supernatant was collected from the cholinergic neuronal differentiated dishes, and the concentration of acetylcholine was measured using Amplex^®^ RED Acetylcholine/Acetylcholinesterase Assay kit (Invitrogen^TM^, ThermoFisher Scientific, Waltham, MA, USA) according to the manufacturer’s instructions. The Ach was monitored indirectly using 10-acetyl-3,7-dihydroxyphenoxazine (Amplex^®^ Red Reagent), a sensitive fluorogenic probe for H_2_O_2_, which is the end product of oxidized choline. After incubating for 1 h at 20 °C, fluorescence was monitored on a plate reader using excitation and emission wavelengths at 550 nm and 595 nm, respectively. The concentration of Ach using a biochemical fluorescent assay was calculated and presented as µM per mL (µM/mL) of culture supernatant.

### 4.11. In Vivo Cell Transplantation of DF-chN into Experimental Rats with Sciatic-Nerve Defects

DF-chN from hDPSCs-cryo were labelled with the fluorescent lipophilic carbocyanine dye, PKH26 (Sigma-Aldrich), following the manufacturer’s instructions [11,16,23]. Then, the cells were collected, washed three time with DPBS, and thereafter transplanted into experimental rats with sciatic-nerve defects. Ten adult male Sprague-Dawley rats (Charles River, Orient Bio Inc., Sungnam, Korea; aged 8–10 weeks; three for Sham OP group, three for non-cell transplanted negative control group, and four for DF-chN transplanted group) were used for all surgical procedures under approved guidelines set by the GNU-161226-R0074, 02, 01, 2017.

The rats were placed under general anesthesia with a subcutaneous injection of 0.5 μL/g of tiletamine/zolazepam (Zoletils^®^, Virbac, Carros, France) and 0.5 µL/g xylazine (Rompuns^®^, Bayer Korea Ltd., Seoul, Korea), as per previous reports [16,23]. For experiments evaluating sciatic-nerve exposure, resection and cell transplantation in the experimental animals were as per previous reports [11,16]. Briefly, the left side of the sciatic nerve in experimental rats was exposed after sequential dissection of the lateral thigh (Figure 6A). Exposed sciatic nerves were resected into the resulting 5-mm gap (Figure 6B). Both resected nerve ends were sutured and enveloped with biodegradable bovine collagen dura mater (Lyoplant^®^) and 6-0 silk suture material to create a tubule in the resected nerve gap, as previously described [11,16] (Figure 6C). In the cell-transplanted groups, a total of 1 × 10^6^ DF-chN were transplanted into the biodegradable tubule in the resected nerve gap with 0.1 mL of fibrin glue (Greenplast^®^ kit), as previously reported [16]. In the negative control group (Control), only the fibrin glue scaffold was injected into the absorbable nerve tubule in the resected nerve gap. The Sham OP group (Sham OP) included dissection of the lateral thigh in experimental rats with exposed sciatic nerves; however, the nerve was not resected.

### 4.12. Analysis of the Scale of Functional Nerve Regeneration by the Open Field Test (OFT)

At eight weeks after cell transplantation in the resected nerve gap, the experimental animals were analyzed for functional nerve regeneration. The animals’ behavioral activities were evaluated using an open-field test (OFT), which examines the walking ability (velocity: cm/s), distance moved (cm), and number of wall climbs in a specific time interval. The Basso, Beattie, Bresnahan (BBB) locomotor rating scale was used to assess the functional outcome for rat hindlimb motor function. Briefly, rats were kept in an open-field area consisting of a metal circular enclosure (100-cm diameter, 18-cm wall height). The ability of rats to walk the length of the tank, measured as walking speed and distance moved, was assessed by a motion sensor, similar to previous reports [16,38].

### 4.13. Histological Analysis of In Vivo Peripheral Nerve Regeneration

At eight weeks after cell transplantation, and following the OFT behavioral test, animals were euthanized by KCl injection under general anesthesia. The sciatic nerves experimented on were re-exposed (Figure 6D) and harvested en bloc, and the specimens fixed with 10% neutral buffered formalin for 24 h. Thereafter, specimens were embedded in a paraffin block, cut into 4-µm sections, and then mounted on silane-coated slides. Cut sections were maintained at 20 °C for 12 h and then deparaffinized, before hematoxylin and eosin staining was conducted after rehydration. Immunohistochemical (IHC) staining for p75 nerve growth factor receptor (p75NGFR) was conducted using an automated immunostainer (LabVision Autostainer^TM^; LabVision, Thermo Fisher Scientific Inc., Fremont, CA, USA). Deparaffinization and antigen retrieval of specimens was performed simultaneously using Tris-EDTA buffer (LabVision). Glass slides were incubated in a PTmodule^TM^ (LabVision) at 100 °C for 25 min, washed with Tris-buffered saline (LabVision) twice (3 min each), and treated with hydrogen peroxide at 20 °C for 10 min. The primary mouse monoclonal p75NGFR antibody (sc-13577, Santa Cruz) was used at a dilution of 1:200 to detect p75NGFR expression in the regenerative nerve tissue. In brief, tissue sections were incubated with primary antibody at 20 °C for 40 min and then treated with a biotinylated polyvalent secondary antibody solution followed by horseradish peroxidase-conjugated avidin/biotin complex, and finally treated with 3,3′-diaminobenzidine and hydrogen peroxide. The nuclei were counterstained with hematoxylin. Immunostaining was then observed under a light microscope (Nikon DIAPHOT 300, Nikon Instrument). A minimum of three longitudinal nerve sections per animal were evaluated for each experimental group (Control, Sham OP, and Cell Transplanted group) for immunostaining analysis. Specifically, in each experimental animal, a higher magnification of regenerated nerve fibers was observed in the proximal, middle, and distal sites of nerve specimens after gross observation of the regenerated nerve fibers at lower magnification.

The fluorescence intensity of PKH26 was observed as previously described [11,16,23]. Briefly, tissues were embedded in an optimal cutting temperature (OCT) compound (Tissue-Tek^TM^, Sakura Fine technical Co. Ltd., Tokyo, Japan), and rapidly frozen at −23 °C and cut into 4-μm sections using Cryo cut equipment (Leica CM3050S, Leica, Wetzlar, Germany). The sections were mounted on a glass slide and counterstained with 4′,6-diamino-2-phenylindole (DAPI; Vectasheid^TM^, Vector, Burlingame, CA, USA). The glass slide was assessed for PKH26 expression using a fluorescence microscope (BX51, Olympus, Tokyo, Japan) equipped with a fluorescent digital camera (DP72, Olympus).

### 4.14. Statistical Analysis

Independent experiments were repeated a minimum of three times, and the data shown are presented as the mean ± standard deviation (SD). Statistical differences between experimental groups were determined using one-way analysis of variance (ANOVA), followed by a Tukey’s test for multiple comparisons or an unpaired *t*-test for single comparisons of experimental data relative to the control value using the PASW statistics 18 software (SPSS Inc., version 21, Hong Kong). Significance was set at *p* < 0.05, with significant differences denoted by an asterisk (*).

## 5. Conclusions

The results from the present study demonstrate that stem cells derived from long-term cryopreserved dental pulp tissues (hDPSC-cryo) display the same MSC characteristics compared to those from fresh dental pulps. Moreover, hDPSCs-cryo showed successful in vitro differentiation potential into cholinergic neurons (DF-chN), which was confirmed through the abundant expression of cholinergic neuron-specific markers at both the mRNA and protein levels. In vivo transplantation of DF-chN in experimental rats with sciatic-nerve defects showed a remarkable enhancement of motor nerve regeneration, demonstrating notably improved behavioral activities using an OFT, and enhanced regeneration of axonal fibers using IHC. Importantly, this study indicates that the cryopreservation of dental pulp tissues offers a useful resource for autologous cells in the nerve regeneration field, including cholinergic nerves.

## Figures and Tables

**Table 1:** List of primers used for evaluating of in vitro differentiations of human dental pulp tissues harvested from extracted wisdom teeth (hDPSCs) into mesenchymal lineages and cholinergic neurons.

Gene	Primer Sequences	Product Size (bp)	Annealing Temp (°C)	Accession No.
*RUNX2*	F: 5′-ATGTGTGTTTGTTTCAGCAG-3′	199	60	NM_001024630.3
	R: 5′-TCCCTAAAGTCACTCGGTAT-3′			
*ON*	F: 5′-GTGCAGAGGAAACCGAAGAG-3′	202	60	J03040.1
	R: 5′-AAGTGGCAGGAAGAGTCGAA-3′			
*BMP2*	F: 5′-TAGACCTGTATCGCAGGCAC-3′	149	60	NM_001200.2
	R: 5′-GGTTGTTTTCCCACTCGTTT-3′			
*PPARγ2*	F: 5′-TGCTGTCATTATTCTCAGTGGA-3′	124	60	AB565476.1
	R: 5′-GAGGACTCAGGGTGGTTCAG-3′			
*FABP4*	F: 5′-TGAGATTTCCTTCATACTGGG-3′	128	60	NM_001442.2
	R: 5′-TGGTTGATTTTCCATCCCAT-3′			
*LPL*	F: 5′-AGACACAGCTGAGGACACTT-3′	137	60	NM_000237.2
	R: 5′-GCACCCAACTCTCATACATT-3′			
*SOX9*	F: 5′-ATGGAGCAGCGAAATCAACG-3′	118	60	BC007951.2
	R: 5′-CAAAGTCCAAACAGGCAGAGAG-3′			
*AGGRECAN*	F: 5′-GAATGGGAACCAGCCTATACC-3′	98	60	NM_001135.3
	R: 5′-TCTGTACTTTCCTCTGTTGCTG-3′			
*COLLAGEN II*	F: 5′-GAGACCTGAAACTCTGCCACC-3′	165	55	NM_001844.4
	R: 5′-TGCTCCACCAGTTCTTCTTGG-3′			
*ChAT*	F: 5′-AGCAGAAATGCAGCCCTGAT-3′	182	60	NM_001142933.1
	R: 5′-GTCAGTCACGGCTCTCACAA-3′			
*HB9*	F: 5′-CAAGCTCAACAAGTACCTGT-3′	134	56	NM_001165255.1
	R: 5′-GCTCTTTGGCCTTTTTGCTG-3′			
*ISL1*	F: 5′-TGGTCATTGCCTTGCCAAAC-3′	106	56	XM_011543380.2
	R: 5′-TCAAACCAATGCAGCTCCAC-3′			
*YWHAZ (reference)*	F: 5′-CTTCACAAGCAGAGAGCAAAG-3′	102	55	NM_003406.3
	R: 5′-CGACAATCCCTTTCTTGTCATC-3′			

Abbreviation: F, forward primer sequence; R, reverse primer sequence; RUNX2, runt-related transcription factor 2; ON, osteonectin; BMP2, bone morphogenetic protein 2; PPARγ2, peroxisome proliferator activated receptor- γ2; FABP4, fatty acid binding protein 4; LPL, lipoprotein lipase; SOX9, sex determining region Y-box 9; AGGRECAN, cartilage-specific proteoglycan core protein; COLLAGEN II, type II collagen; ChAT, choline acetyltransferase; HB9, homeobox HB9; ISL1, insulin gene enhancer protein ISL-1; YWHAZ, Tyrosine 3-monooxygenease/ tryptophan 5-monooxygenease activation protein, zeta polypeptide.

## Abbreviations

MSCs	Mesenchymal stem cells
hDPSCs-cryo	Stem cells (MSCs) from cryopreserved human dental pulp
hDPSCs-fresh	Stem cells (MSCs) from fresh human dental pulp
D609	Tricyclodecane-9-yl-xanthogenate
DF-chN	Differentiated cholinergic neurons
p75NGFR	Low-affinity nerve growth factor receptor
ChAT	Choline acetyltransferase
Ach	Acetylcholine
IHC	Immunohistochemistry or Immunohistochemical
Oct4	Octamer-binding transcription factor 4
Sox2	Sex determining region Y-box 2
FABP	Fatty-acid binding protein
LPL	Lipoprotein lipase
PPARγ	Peroxisome proliferator-activated receptor gamma
ON	Osteonectin
RUNX2	Runt-related transcription factor 2
BMP2	Bone morphogenetic protein 2
COLLAGENII	Type II collagen
SOX9	Sex determining region Y-box 9
HB9	Motor neuron and pancreas homeobox 1, MNX1
ISL1	Insulin gene enhancer protein ISL-1
PC-PLC	Phosphatidylcholine-specific phospholipase C
